# Syncopes, paresis and loss of vision after COVID-19 mRNA-based vaccination and SARS-CoV-2 infection

**DOI:** 10.1007/s15010-024-02439-y

**Published:** 2024-12-02

**Authors:** Tobias Weirauch, Gundolf Schüttfort, Maria J. G. T. Vehreschild

**Affiliations:** Goethe University Frankfurt, University Hospital Frankfurt, Department II of Internal Medicine, Infectious Diseases, Theodor-Stern-Kai 7, 60590 Frankfurt, Germany

**Keywords:** COVID-19, mRNA-based vaccines, Post-Vac-syndrome, Post-COVID-19-syndrome

## Abstract

mRNA-based vaccines played a key role in fighting the global COVID-19 pandemic by saving millions of lives. In rare cases, however, the BNT162b2 vaccine has been associated with severe adverse reactions e.g. myocarditis (OE ratio 2.78; 95% CI 2.61; 2.95) [Faksova in Vaccine 42(9):2200-2211, 2024, 10.1016/j.vaccine.2024.01.100, Schwab in Clin Res Cardiol 112(3):431-440, 2022, 10.1007/s00392-022-02129-5]. Here, we describe the case of a 38-year-old man who developed a wide variety of long-term symptoms (fatigue, dizziness, palpitations with recurrent syncopes, paresthesia, paresis and fasciculations) following his first mRNA-based BNT162b2 COVID-19 vaccination. 143 days after vaccination, a subsequent COVID-19 infection was associated with exacerbation of paresis and a temporary loss of vision. After ruling out other causes and due to the immediate temporal association, an adverse reaction to vaccination appears likely. The fact that these symptoms worsened after a subsequent acute COVID 19 infection hints at the possibility of a common underlying pathophysiology. This case combines two clinical phenomena that have emerged during the COVID 19 pandemic, side effects associated with novel vaccines and Post-COVID Syndrome.

## Introduction

COVID-19 is a potentially lethal systemic disease caused by SARS-CoV-2. The virus is transmitted to humans primarily via respiratory droplets [3]. The overall case fatality rates (CFR) range from 0.27 to 1.0% [4, 5]. In 2020, coughing (86.1%), fever (85.0%) and shortness of breath (80.0%) were the most common COVID-19 symptoms at hospitalization, which however, was largely influenced by underlying conditions such as hypertension (49.7%), obesity (48.3%), chronic lung disease (34.6%), diabetes mellitus (28.3%), and cardiovascular disease (27.8%) [6]. In addition, recent work on risk factor identification points out the importance of non-white/european ethnicity and virus variants [7]. In response to the COVID-19 pandemic, a broad range of new vaccines were developed and over 13 billion COVID-19 vaccine doses administered. Especially the new mRNA-based vaccine technology gained enormous importance in combating the pandemic. Mortality and excess mortality from 185 countries globally were used to estimate the number of deaths averted by vaccination. Using a mathematical model, this study estimates that the global vaccination campaign prevented more than 14.4 million deaths in the first year of vaccination [8]. In very few individual cases, however, patients experienced significant complications such as myocarditis, stroke, thrombosis or long-term impairments [9]. Evaluating potential associations between vaccination and development of novel signs and symptoms can be challenging but should be considered in cases where the following criteria apply: i) no other cause for the signs and symptoms can be identified through in-depth diagnostics and ii) there is temporal association with vaccination.

Here, we describe a rare case of multiple and longlasting symptoms in potential association with a mRNA-based vaccine, which were subsequently exacerbated by a COVID-19 infection.

## Case history

A 38-year-old man (BMI 21 kg/m^2^) with no medical history received his first BNT162b2 (Comirnaty) mRNA-based vaccine from BioNTec/Pfizer in September 2021. Six hours after vaccination, the patient developed severe cephalgia and dizziness lasting for several days. Recurrent syncopes (up to 4–6 times per day) occurring in combination with palpitations emerged two days after vaccination and led the patient to visit the emergency unit, where a syncope was testified by medical staff. Spontaneous syncope occurred 20–30 times over the following two weeks. Even though the syncopes disappeared, the patient continued to observe irregular heartbeats. In addition, he reported new onset of fatigue, brain fog, acral paresthesia, paresis of forearm extensors and Mm. quadriceps femoris, disseminated fasciculations and urticaria in the weeks following the vaccination. With the exception of the syncopes, all symptoms persisted in varying degrees of severity. Overall, the severity of symptoms decreased slightly over the following months. He received a cranial MRI and a cardiac MRI. Both imaging modalities did not yield any abnormal findings (Table 1).

**Table 1:** Summary of diagnostic findings

	Day after vaccination	Summary of findings
Electrocardiogram	Day 2	- Sinus rhythm (60/min) - PQ 150 ms, regular QRS, regular R progression, regular ST segment
Long-term Electrocardiogram	Day 20	- Sinus rhythm (frequency spectrum: 51 to 137/min; average: 81/min) - No ventricular extrasystole - No arrhytmia - No relevant cardiac rest or AV-block - Single AV-block (Wenckebach) during night
Echocardiogram	Day 20	- Regular atria and ventricle diameter - Regular heart function - Regular doppler sonography - No morphological valve findings - No restrictions of wall movement - No pericardial effusion
Long-term blood pressure	Day 24	- Physiological blood pressure without antihypertensive drug intake - Physiological drop of blood pressure and heart beat during night - No pathological hyper- or hypotensive findings
Chest CT	Day 97	- No lymphadenopathy - Regular heart and vessel structures - No pericardial effusion - No pleural effusion - No infiltrations - No bone abnormalities
Cardiac MRI	Day 121	- Regular heart size, structure, valves and perfusion - No myocardial ischemia, necrosis or fibrosis - No ventricle dilatation - No edema or inflammation
Nerve conduction velocity	Day 125	- N. tibialis: right MSAP 23,2 mV, NCV 41,1 m/s; left MSAP 20,1 mV, NCV 42,7 m/s - N. suralis: right SNAP 19,2 μV, NCV 42,9 m/s; left SNAP 19,1 μV, NCV 44,7 m/s
Cranial MRI	Day 157	- No restrictions of brain diffusion - No permeability dysfunction - No suspect areas of brain parenchyma - No morphological brainstem changes - No signs of acute inflammation - Deviation of nasal septum
Ophthalmological	Day 176	- Moderate astigmatism and hyperopia - Regular intraocular pressure - Regular retinal vessel caliber - No retinopathy - No signs of inflammation

143 days after vaccination, the patient suffered a PCR confirmed SARS-CoV-2 infection, and the symptoms experienced after vaccination exacerbated. His most debilitating symptom presented as severe paresis of arms and legs, blurred vision, and a temporary loss of vision. Frequency, intensity, and duration of symptoms varied over the course of the following year. During the COVID-19 infection, the patient also presented with fever (38.4 °C) and SARS-CoV-2 associated respiratory symptoms, such as cough and a sore throat, which lasted only for a few days. Even months after the infection, newly observed headache and visceral pain persisted in varying degrees and severity. As of March 2024, a mild paresis and fatigue persist, and episodes of blurred vision still occur occasionally.

**Fig. 1:**
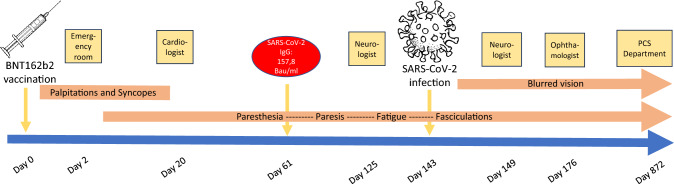
Timeline of vaccination and COVID-19 associated side effects

Various specialists tried to clarify the origin of the symptoms. Unfortunately, the specialist medical workup was initiated only after several weeks (Fig. 1). Neither a comprehensive cardiopulmonary evaluation (echocardiogram, stress and long-term ECG, long-term blood pressure measurement, cardio MRI) nor a neurological assessment (intensive clinical assessment excluding multiple sclerosis and Guillain-Barré-Syndrome, cranial CT, nerve conduction velocity (NCV)) were able to link the symptoms to a conclusive diagnosis. An ophthalmological examination with intraocular pressure assessment, slit lamp and fundus examination revealed astigmatism and hyperopia, but no compatible findings. The results are summarized in Table 1.

A single ventricular premature beat as well as a second-degree atrioventricular block (Wenckebach) were found in a long-term ECG four weeks after vaccination. However, a cause of an inadequate cardiac transmission that could explain the previous severity of syncopes was not found. Due to the time delay between symptom onset and thorough cardiological diagnostics, it remains unknown which cardiac aspects were underlying.

The clinical neurological examination showed abnormal findings and side differences in grade of strength (left forearm extensors 3/5, left Mm. quadriceps 4/5), muscle reflexes and muscle tone. Reflexes (biceps-, triceps-, patellar- and achilles tendon) were weak in the left upper and lower body. Approximately five fasciculations of left forearm extensors occurred at irregular intervals during 30 min of examination. However, neither the NCV (no pathological findings) nor the cranial MRI (no morphological alterations) could identify the origin of the symptoms (Table 1).

No relevant laboratory results including electrolyte abnormalities could be found shortly after vaccination or during the COVID-19 infection. There was no increase in CRP, d-dimers, troponine or an electrolyte imbalance at either time. Blood count, transaminases, creatinine, creatine kinase and TSH remained normal over the 2.5 year follow up. No evidence of other chronic infectious diseases was found. The screening excluded HIV, neurosyphilis, gonorrhea, tuberculosis, Lyme disease, intracellular bacteria (Chlamydia, Coxiella, Rickettsia), reactivation of CMV and EBV as well as hepatitis A, B and C. SARS-CoV-2 IgG was 157,8 Bau/ml two months after vaccination. Furthermore, the performed serological (cANCA, pANCA, ANA), radiological and various additional investigations revealed no evidence of a rheumatologic pathogenesis of the symptoms.

## Discussion

In this report, we present a case with symptoms suggesting prolonged reactions to BNT162b2 that underlines the potential variability of symptoms, as well as the risk of an exacerbation due to a subsequent COVID-19 infection.

Cardiac related syncopes [10–12] and myocardial damage in timely association with this vaccine have been previously reported [2, 13, 14]. The Paul-Ehrlich-Institute (PEI) in Langen, Germany indicates the BNT162b2-associated risk of syncopes as 0.62 per 100.000 and that of myocarditis as 1.58 per 100.000 [15]. An arrhythmogenic cause of syncope, e.g. as a result of a vaccine-induced myocarditis, is conceivable due to the recorded second-degree atrioventricular block (Wenckebach). The NEURO-COVAX population-based study (n = 19.108) from Salsone et al. described several of our patient`s symptoms, e.g. brain fog (6.4%), dizziness (13.4%) and paresthesia (10.4%), but they typically decrease after a few days [16]. Cutaneous findings like urticaria have also been linked to mRNA-based vaccines in approx. 0.4% [19, 20]. To date, only individual cases of transient loss of vision have been published [19, 20]. Cases with a wide variety of other symptoms including fever, skin rashes, dizziness, brain fog, etc., have been described after mRNA-vaccination [21].

The details on the mechanisms underlying adverse side effects caused by mRNA-based vaccines remain unclear. Our patient, however, reported the onset of his symptoms only six hours after vaccination. While the underlying pathomechanism remains unclear, a mechanism based on an allergic or intolerance reaction would be compatible with this timely association. On the other hand, similar symptoms after vaccinations have been reported before the era of mRNA-vaccines. Additionally, non-mRNA-vaccines have already been associated to a variety of autoimmune processes, as summarized by Guimarães et al. [23].

We were unable to identify any published data on the aggravation of COVID-19 vaccine-related side effects by a COVID-19 infection. The similarity between symptoms of PCS and those of patients being assessed for vaccine-related side effects has come to our attention in clinical practice. It can be hypothesized that both entities are triggered by similar underlying pathomechanism. In line with this hypothesis, aggravation of PCS symptoms after SARS-CoV-2 vaccination has been previously reported [24–27]. At this point, there is, however, no data supporting this hypothesis and further research is warranted.

To conclude, mRNA-based COVID-19 vaccines remain safe and highly effective despite reports of associated cases of severe adverse events. For the people affected by potential mRNA vaccine-associated symptoms, traditional vaccine technologies should be considered for future vaccinations. Further mechanistic research assessing the pathologies that may trigger mRNA vaccine-associated symptoms are urgently needed.

## Limitations

The authors are aware that the small number of objectifiable symptoms represent a limitation. However, we were able to detect pathological findings during the clinical examination. Further limitations arise from the lack of a detailed psychiatric or psychosomatic assessment and thus the possibility that components of the symptoms may have been related to a nocebo-effect. It cannot be ruled out that a cardiac abnormality was already present before the time of vaccination. In addition, some statements are based on the patient’s anamnesis only, which, however, appeared fully reliable.

## Data Availability

No datasets were generated or analysed during the current study.

## Abbreviations

ANA: Antinuclear antibody
ANCA: Anti-neutrophili cytoplasmatic antibody
Approx.: Approximately
BMI: Body mass index
CMV: Cytomegalovirus
CRP: C-reactive protein
CT: Computed tomography
EBV: Eppstein-Barr virus
ECG: Electrocardiogramm
HIV: Human immunodeficiency virus
i.e.: Id est
MRI: Magnetic resonance imaging
MSAP: Motory action potential
Mm.: Musculi
mRNA: Messenger ribonucleic acid
NCV: Nerve conduction velocity
PCR: Polymerase chain reaction
PCS: Post-COVID syndrome
PEI: Paul Ehrlich Institute
PVS: Post-Vac syndrome
SARS-CoV-2: Severe acute respiratory syndrome coronavirus type 2
SNAP: Sensory action potential
